# GERIATRIC WORKFORCE ENHANCEMENT PROGRAM: EDUCATIONAL OUTCOMES OF DEMENTIA CAREGIVER CONFERENCES IN RURAL UTAH

**DOI:** 10.1093/geroni/igz038.3392

**Published:** 2019-11-08

**Authors:** Kara B Dassel, Rand Rupper, Katherine Supiano, Troy Andersen, Jorie Butler, Jacqueline Telonidis, Linda S Edelman

**Affiliations:** University of Utah, Salt Lake City, Utah, United States

## Abstract

The University of Utah was awarded federal funds to establish a Geriatrics Workforce Enhancement Program (GWEP) focused on integrating geriatric training into 20 long-term care centers across the state of Utah. One specific objective of the GWEP was to: Provide community-based dementia education to 500 community participants from four geographically diverse urban and rural locations. In year four of the project, an interdisciplinary academic and community-based team planned and implemented dementia caregiver conferences in two rural areas in Utah that included American Indian (AI) tribal communities. Educational topics included an overview of dementia, engaging in goals of care discussions, managing caregiver stress, and finding local resources. Demographic surveys and pre-post modified 15-item version of the Alzheimer’s Disease Knowledge Scale (ADKS; Carpenter et al., 2009) were administered. A total of 148 participants attended the conferences. Participants were primarily female (76.6%), white (84.2%), and self-identified as family/informal caregivers (35.0%). Of the participants, 17.8% self-identified as AI. Overall, there were no significant differences on the pre- and post ADKS scores (mean scores of 12.45 and 12.5 out of 15 possible points, respectively). However, AI ADKS mean scores were lower (10.2 and 10.5, respectively; range 9-13) than white participants (12.7 for both, respectively; range 12-13) reflecting a greater need for ADRD education in AI tribal communities. The conferences were well-received with 82.5% of attendees said that the conference met their educational needs and 83.9% reported that the information would improve the type of care they provide to someone with dementia.

